# *Toxoplasma*-induced changes in host risk behaviour are independent of parasite-derived AaaH2 tyrosine hydroxylase

**DOI:** 10.1038/s41598-017-13229-y

**Published:** 2017-10-23

**Authors:** Cristina Afonso, Vitor B. Paixão, Andreas Klaus, Matteo Lunghi, Federica Piro, Carla Emiliani, Manlio Di Cristina, Rui M. Costa

**Affiliations:** 1 Champalimaud Center for the Unknown, Champalimaud Neuroscience Programme, Av. Brasília, Doca de Pedrouços, 1400-038 Lisboa, Portugal; 2 University of Perugia, Department of Chemistry, Biology and Biotechnology, Building B, Via del Giochetto, 06122 Perugia, Italy

## Abstract

*Toxoplasma gondii* infects a broad range of hosts and can establish chronic infections with the formation of brain cysts. Infected animals show altered risk behaviour which has been suggested to increase capture probability of hosts, and thus enhance parasite transmission. It has been proposed that the ability of *Toxoplasma* cysts to secrete tyrosine hydroxylase could mediate these behavioural alterations. We tested the involvement of secreted tyrosine hydroxylase, coded by the parasite *AaaH2* gene, in the development of alterations in mouse behaviour, by generating an *AaaH2* deletion mutant parasite strain and testing its influence on behaviour. We found that both mice infected with wild type or *AaaH2* mutant strains showed changes in risk behaviour. We confirmed these findings using factor analysis of the behaviour, which revealed that behavioural changes happened along a single dimension, and were observed in both infected groups. Furthermore, we developed a new behavioural paradigm in which animals are unpredictably trapped, and observed that both groups of infected animals perceive trapping but fail to adjust their behaviour to avoid further trapping. These results demonstrate that parasite-secreted AaaH2 TH is neither necessary for the generation of risky behaviour nor for the increased trappability observed during chronic *Toxoplasma* infection.

## Introduction

The protozoan parasite *Toxoplasma gondii* has the ability to infect numerous animals, such as mammals (including humans) and birds. Infection by this organism is one of the most predominant in the world^1^ and, because of its wide host range, this parasite is generally considered as one of the most successful pathogens among eukaryotes. The parasite’s life cycle is complex, involving two types of hosts: definitive and intermediate. Felines are the only definitive hosts and sexual parasitic reproduction happens exclusively in this animal group, resulting in contamination of the environment after excretion of infective *Toxoplasma* oocysts in feline faeces^2^. The majority of parasite propagation happens within the intermediate host population, by consumption of infected plant food (herbivores) and meat (carnivores) or by vertical transmission from mother to progeny. Once inside a new host, the parasite multiplies asexually into freely moving tachyzoites. These will infect various cell types (neurons being one of the most frequent) and differentiate into bradyzoite-containing cysts, which persist chronically^3^.

Mice and rats are naturally infected by this parasite and infection results in cyst formation in rodent brains and production of a number of modifications in these hosts’ behaviour (reviewed in ref.^4^). These modifications are diverse and range from reduced avoidance of feline odour^5^ to increased activity^6^ and trappability^7^, and also changes in the microstructure of exploratory behaviour and risk/unconditioned fear^8,9^. These changes would impact normal defensive responses of the host and, because of this, it has been proposed that they are beneficial for the parasite, underlying the increase in capture probability described in rodents^7^. This would then result in facilitation of parasite transmission to subsequent hosts, thus arguing for parasite manipulation of host behaviour. Additionally, studies have shown that this parasite depends mainly on clonal expansion within the intermediate host animal group rather than sexual reproduction in the definitive cat host^10^. This highlights the evolutionary importance of parasite transmission between intermediate hosts.

The consistent occurrence of brain cysts together with the simultaneous emergence of changes in normal behaviour raises the possibility that the presence of the parasite could produce changes in neuronal activity. One possible way to modulate neuronal activity is by acting directly on neurotransmitter metabolism, and a growing body of evidence supports the ability of parasites to manipulate host dopamine levels. An original study first described an increase in dopamine levels in *Toxoplasma*-infected mouse brains^11^, presumably in a cell-autonomous way since *in vitro* infection of dopaminergic cells (PC12) results in increased dopamine synthesis and release^12,13^. Control of host cell dopamine synthesis could derive from the parasite, as analysis of *Toxoplasma gondii* genome identified 2 genes (*AaaH1* and *AaaH2*) encoding tyrosine hydroxylase (TH), which contains a N-terminal secretion peptide^14^. This enzyme is involved in catalysing the conversion of L-tyrosine into dopamine’s precursor molecule L-dihydroxyphenylalanine (L-DOPA). The *AaaH1* gene is constitutively expressed in both tachyzoites and bradyzoites whereas *AaaH2* expression is uniquely upregulated in bradyzoites (cysts)^14^. Together, these observations raise the hypothesis that AaaH2 TH can modulate host dopamine levels during chronic infection, and that this modulation affects host behaviour.

Parasite-derived TH has been observed outside individual bradyzoites but still inside cysts^12^, most likely as a consequence of export driven by the secretion peptide. Furthermore, a recent study has reported that host cell DOPA decarboxylase (DDC), the enzyme that uses L-DOPA as a substrate to directly produce dopamine, is also recruited to tissue cysts^13^. This ability of the parasite to produce/recruit the two enzymes necessary for dopamine production further supports the possibility of *Toxoplasma*-driven synthesis of additional dopamine. Additional support for the hypothesis that infection results in increased dopamine levels in the host comes from the changes in behaviour observed in mice where the dopamine transporter DAT has been deleted^15^. These mutant mice show high levels of extracellular dopamine and display behaviour changes similar to the ones observed during *Toxoplasma* chronic infection (increased locomotor activity, lack of habituation to novel environment). However, reports of the effects of *Toxoplasma* infection on dopamine metabolism are not without controversy. In at least three independent studies^16–18^, authors failed to observe an effect on host dopamine levels or reported a reduction in this neurotransmitter levels^19^ in chronic parasitic infection. Furthermore, neither overexpression nor deletion of the *AaaH2* gene from infective parasites had any impact on dopamine levels *in vitro* or *in vivo*^18^. Regardless of whether parasites can affect host dopamine levels through *AaaH2*-driven TH production, it still remains to be determined whether this enzyme contributes to the production of the host behavioural changes.

The aim of the present study was to investigate whether the *AaaH2* gene, via secretion of tyrosine hydroxylase, is necessary for the establishment of *Toxoplasma*-induced behavioural modifications. To address this, we generated a new strain of *Toxoplasma* where the *AaaH2* gene was deleted, and compared the behaviour of mice chronically infected with the *AaaH2*-deletion strain and its parental counterpart. We observed that both parental and mutant strains produced changes in host behaviours related to risk/exploration, in two different setups, with no substantial differences observed between infected groups. We confirmed these findings by performing factor analysis on the behavioural variables measured, showing that behavioural changes happen along a single dimension, with no distinction between infected groups.

Additionally, we investigated whether some of these changes in behaviour stemmed from an inability of infected animals to perceive environmental risk or rather from a failure to activate defensive behavioural programs. We developed a new behavioural paradigm, in which animals were unpredictably trapped and we compared individual performance before and after this environmental challenge. We show for the first time that *Toxoplasma*-infected animals were capable of perceiving the trapping event, but were not able to adapt their behaviour accordingly; this was observed irrespective of animals being infected with parental or mutant strains.

Taken together, these results indicate that parasite AaaH2 TH is not necessary for the generation of the behaviour changes observed in *Toxoplasma* chronic infection.

## Results

### AaaH2 TH mutant parasite exhibits the same infection dynamics as the parental strain

**Figure 1:**
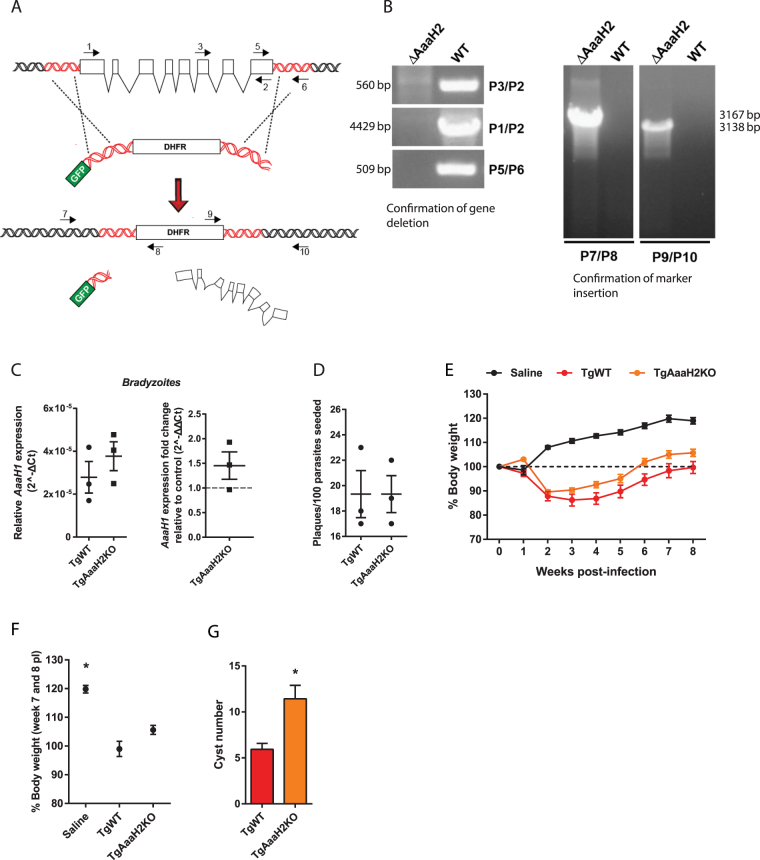
Host infection dynamics remain unaffected after deletion of the *AaaH2* gene from the *Toxoplasma* genome. (**A**) The full *AaaH2* gene sequence (white boxes) was substituted by the coding sequence for the dihydrofolate reductase protein (DHFR). Arrows indicate primer identification and orientation and red sequences represent homology region. (**B**) PCR analysis of purified genomes from mutant parasites (∆*AaaH2*) confirmed deletion of the *AaaH2* gene (left panel), and the presence of the DHFR marker (right panel). The primer pairs used and sizes of the amplification products are indicated. Relevant cropped regions of the gels are shown and full-length gels are presented in Supplementary Figure S3. (**C**) No differences in relative *AaaH1* expression levels were observed in TgWT and TgAaaH2KO bradyzoites (left panel). When compared with control expression levels (TgWT, value of 1), no significant *AaaH1* expression fold change was detected in mutant bradyzoites (right panel). (**D**) No changes in infection efficiency or parasite viability were found between TgAaaH2KO and the parental strain TgWT. (**E**) Weight analysis throughout infection showed that acute infection resulted in marked weight loss over the first 2 weeks. As chronic infection is established, infected animals (independent of infective parasite strain) recover to initial weight values (dotted line), which are always inferior to weight values in control animals (**F**). Mouse saline group, n = 28; TgWT group, n = 15; TgAaaH2KO group, n = 25. (**G**) The number of cysts formed in brains infected with the TgAaaH2KO strain was increased relative to the parental strain (TgWT). Mouse TgWT group, n = 14; TgAaaH2KO group, n = 14. *p < 0.05. The data are presented as the mean ± SEM.

To determine whether parasite-derived AaaH2 TH was involved in the behaviour modifications induced by *Toxoplasma* infection described in previous reports (reviewed in ref.^4^), we generated a mutant version of the parasite, in which the *AaaH2* gene is absent. This involved a targeted replacement of the entire gene by the coding sequence for dihydrofolate reductase (DHFR), a protein which confers resistance to the drug pyrimethamine (Fig. 1A and Materials and Methods). This strategy used the parental strain ME49^*BAG1/EGFP*^ as background (referred to as TgWT). Subsequent PCR analysis confirmed that one parasite clone (TgAaaH2KO) had both the *AaaH2* gene deleted (Fig. 1B, left panel) and replaced by the DHFR marker (Fig. 1B, right panel).

Since TH is also encoded by an additional gene (*AaaH1*)^14^, it’s theoretically possible that a functional compensation occurs in parasites where the *AaaH2* gene has been deleted. This would be reflected as an upregulation of the *AaaH1* gene in the mutant parasite strain. To determine whether *AaaH1* gene expression was altered in the mutant parasite strain, we performed qPCR experiments on differentiated bradyzoites (cyst stage parasite) and found no alteration in *AaaH1* expression in TgAaaH2KO bradyzoites (Fig. 1C).

Additionally, a plaque assay revealed that no differences were found in terms of infection efficiency and parasite viability between TgAaaH2KO and the parental strain TgWT (Fig. 1D). We then used tachyzoites to intraperitoneally infect C57BL/6 J female mice and followed the infections for 8 weeks. Infected animals (irrespective of the infective parasite strain) displayed acute weight loss over the first 2 weeks (Fig. 1E), whereas non-infected animals (saline group) did not. Over the course of the following weeks, infected animals recovered to close to their pre-infection weights, but always weighed significantly less than control animals (Fig. 1F).

The presence of cysts formed after infection with TgWT and TgAaaH2KO strains was also investigated (Fig. 1G). Entire infected brains were sliced, analysed and cysts were found in both TgWT and TgAaaH2KO-infected animals, with no reduction in cyst numbers between infected brains and even an observed increase.

These data demonstrate that chronic infection with TgAaaH2KO strain still displays the same overall dynamics as the wild type (parental) strain (TgWT) and thus, parasite AaaH2 TH is not necessary for acute or chronic infection in C57BL/6 L mice.

### Parasite-derived AaaH2 TH is not necessary for exploratory locomotion changes in infected animals

**Figure 2:**
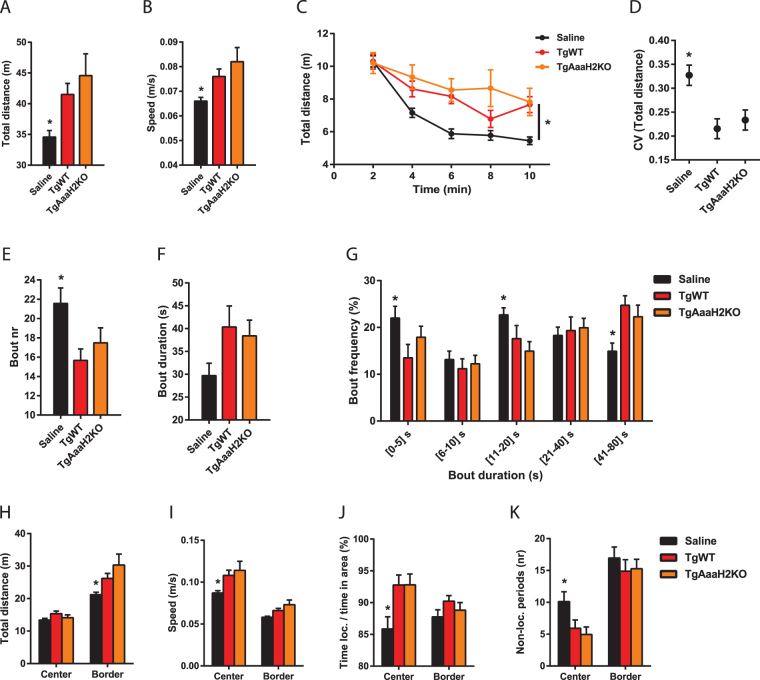
Changes in exploratory behaviour in the open field after chronic *Toxoplasma* infection are independent of parasite AaaH2 TH. (**A**) When compared to controls, both TgWT- and TgAaaH2KO-infected animals showed an increase in the total distance travelled. (**B**) Infected mice moved at higher speeds than control mice. (**C**) Relative to controls, a similar lower ability to habituate to the novel environment was observed in infected animals, as shown by the smaller reduction in locomotion over time. (**D**) Infected animals displayed comparable reduced individual variability in distance covered, relative to controls. (**E**) An equivalent reduction in the number of movement bouts in both infected groups was observed relative to control animals. (**F**) All animals executed movement bouts of comparable duration, as no difference was detected between experimental groups. (**G**) Bout structure was similarly modified in infected mice when compared with controls, with a reduction in the frequency of short bouts and an increase in long bout frequency. (**H**) Infected mice (irrespective of infective strain) covered higher distances in the border, relative to control mice. (**I**) Mice in both infected groups moved faster in the center zone than controls. (**J**) In contrast to control animals, when in the center, infected mice were more frequently engaged in locomotion. (**K**) Contrary to what was observed in controls, infected animals showed a reduction in the number of non-locomoting periods in the center area. (**A**–**K**) Mouse saline group, n = 28; TgWT group, n = 15; TgAaaH2KO group, n = 25. *p < 0.05. The data are presented as the mean ± SEM.

To determine whether parasite AaaH2 TH is involved in producing the alterations in exploratory behaviour previously described by our group during *Toxoplasma* infection^9^, we infected animals with individual parasite strains and compared their performance in an open field (OF). Animals were placed in the center of a square open arena, which constituted a novel environment with exposed and non-exposed zones (center and border, respectively) and their behaviour was recorded for 10 min. This test revealed that, similar to what was observed in TgWT-infected animals, mice infected with the mutant parasite strain travelled longer distances relative to the control group (Fig. 2A), and at higher speeds (Fig. 2B). Additionally, contrary to what was observed in the control group (where there was a marked reduction in locomotion over the test period (habituation)), both groups of infected animals displayed a smaller decrease in locomotion and failure to habituate to the environment (Fig. 2C). This was reflected also in the reduction of individual, minute-to-minute performance variability (distance travelled), as indicated by lower coefficients of variation (CV) for both infected experimental groups, relative to controls (Fig. 2D).

Previous work has suggested that an increase in locomotion during chronic *Toxoplasma* infection was reflected in an altered structure of exploratory movement^9^, so we investigated whether this aspect was also altered in TgAaaH2KO-infected animals. The number of movement bouts displayed by both infected groups was reduced relative to controls (Fig. 2E), with no difference in bout duration (Fig. 2F). Bout frequency analysis also demonstrated that TgAaaH2KO-infected animals organized exploratory locomotion in the same way as TgWT-infected mice. This was characterised by a decrease in the number of short bouts in parallel with an increase in longer duration bouts (Fig. 2G).

The results described above show that the major alterations in locomotion observed in chronically infected C57BL/6 L mice (increased locomotion, higher speeds, reduced habituation and performance variability, and a re-organization of movement structure in a novel environment) are independent of parasite-secreted AaaH2 TH.

### *Toxoplasma*-induced changes in exploration in exposed/non-exposed areas are not dependent on AaaH2 TH

As described in our previous study, the changes in movement structure extend to the two specific zones that can be defined in the OF (center - exposed *versus* border – non-exposed), with infected animals behaving differently from controls when in these areas^9^. To investigate whether the absence of AaaH2 TH in the infective parasites would impact this area distinction, we analysed the spatial distribution of locomotion. We found that C57BL/6 L infected mice (irrespective of the infective strain) displayed increased locomotion in the border (Fig. 2H) and, when in the center area, moved at higher speeds (Fig. 2I) compared to controls. Center area behaviour was also distinct in these animals in that they spent more time engaged in locomotion (Fig. 2J), and also showed reduced number of non-locomoting periods (episodes of no horizontal movement, including grooming, rearing, etc) (Fig. 2K).

Overall, these results indicate that the particular behaviour of infected animals in distinct areas of the open field does not depend on parasite-derived AaaH2 TH.

### Parasite-induced changes in the response to exposed areas are independent of AaaH2 TH

The results above describe a differential behaviour of infected animals in exposed areas, which might reflect differences in the ability to asses risk or in fear responses. To address this, we used the elevated plus maze (EPM) to probe for alterations in fear/risk responses (reviewed in^20^ and used in^9^). This test takes advantage of mice’s innate fear of open, potentially unsafe spaces (open arms, OA) and highlights a bias for occupation of enclosed, protected areas (closed arms, CA).

**Figure 3:**
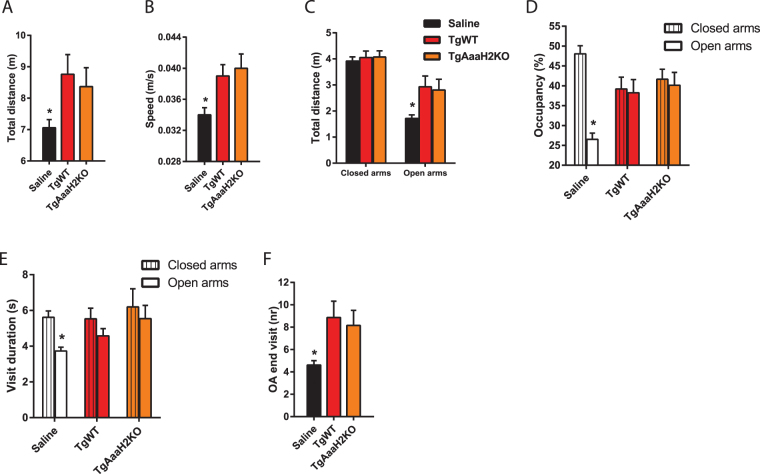
Modifications in host fear-related behaviour in the elevated plus maze are independent of AaaH2 TH in the parasite. (**A**) Infected animals exhibited a similar increase in the distance covered in this setup, in comparison to controls. (**B**) Speed values were equally higher in both infected groups, relative to controls. (**C**) Infected mice were able to cover longer distances in the open arms than control mice. (**D**) Control animals preferred closed arms over open arms whereas animals in both infected groups did not show this occupancy bias. (**E**) Visit duration to closed arms was increased in controls and this difference was not detected in infected mice. (**F**) Differently from controls, infected animals showed a higher number of visits to the extremity of the open arms. (**A**–**F**) Mouse saline group, n = 28; TgWT group, n = 15; TgAaaH2KO group, n = 25. *p < 0.05. The data are presented as the mean ± SEM.

Similar to what was observed in the OF test, TgAaaH2KO-infected animals’ behaviour in the EPM was indistinguishable from that of TgWT-infected mice. Animals in both groups showed higher and faster locomotion (Fig. 3A and B) relative to controls. The observed increased locomotion derived mainly from higher locomotion values in the open arms (Fig. 3C). Additionally, the expected occupancy bias between closed and open arms was present in controls, with animals preferring CA over OA (Fig. 3D). This preference was absent in both infected groups (Fig. 3D) and also reflected in equal duration of visits to the two arm types (Fig. 3E). Finally, infected animals travelled more often to the most distal part of the open arms, in a way that can be considered highly risky/fearless (Fig. 3F).

These data indicate that risky behaviour/unconditioned fear responses observed in C57BL/6 L infected animals are not affected by the absence of the parasite-derived AaaH2 TH.

### Changes in behaviour are organized along specific dimensions

**Figure 4:**
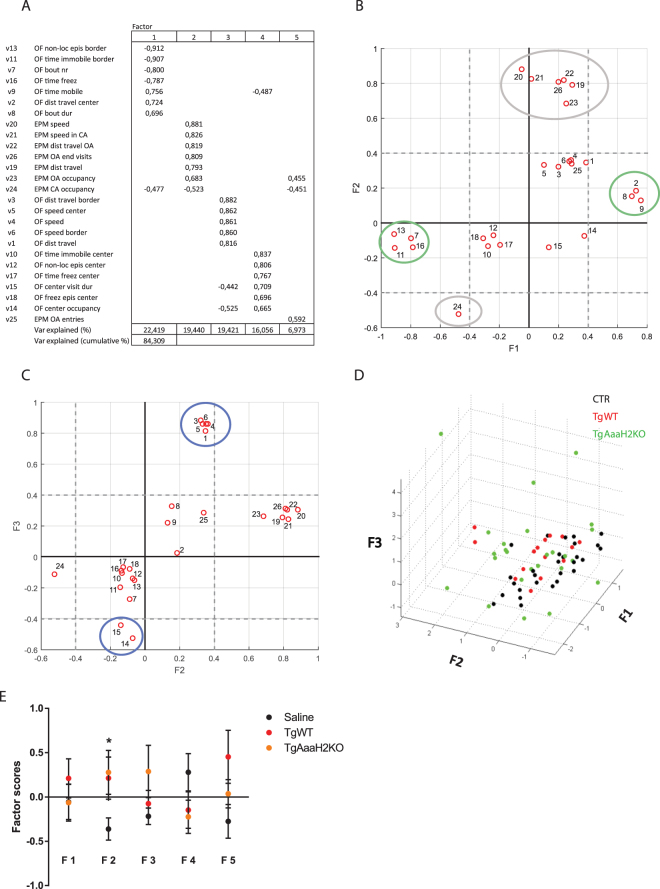
Overall behavioural changes are structured along specific dimensions. (**A**) Main factors (and respective loadings) obtained following factor analysis of behavioural variables (v1-v26) measured in the open field (OF) and elevated plus maze (EPM). (**B**,**C**) Distribution of the behavioural variables (1–26) in bi-dimensional spatial projections of the first three dimensions (Factors 1, 2 and 3). Coloured ellipses represent different variable groupings (see text for details). (**D**) Spatial distribution of individuals of the different experimental groups in the tridimensional space defined by Factors 1, 2 and 3. Mouse saline group, n = 25; TgWT group, n = 15; TgAaaH2KO group, n = 25. (**E**) Factor scores for individual animals in the three experimental groups calculated from loadings derived from factor analysis. Although no differences were detected between TgWT and TgAaaH2KO-infected animals, both infected groups showed significantly different loadings, relative to controls, for Factor 2, which represents the structure of risk/unconditioned fear behaviour. *p < 0.05. The data are presented as the mean ± SEM.

To reduce the complexity of the collected behavioural data and reveal possible hidden data structure, we used factor analysis. This allowed us to gain an unbiased insight into the behavioural dimensions that were being modified during chronic infection with TgWT parasite strain and its AaaH2 TH mutant counterpart. We identified five factors (dimensions) which represented the primary behavioural parameters being measured in the OF and EPM tests. These five factors accounted for 84.3% of the variance present in the data and factor loadings are depicted in Fig. 4A. The factor that explained the highest variance percentage (Factor 1, 22.4%) was negatively loaded by variables related to the absence of horizontal displacement and structure of movement. Factor 1 also contained positive loadings in variables that described general locomotion and thus this factor may reflect the structure of exploration/locomotion. The second extracted factor (Factor 2) accounted for 19.4% of the variance and was positively loaded exclusively by variables that represented features measured in the EPM test. Therefore, we considered that this factor described alterations in risk/unconditioned fear behaviour. The third factor (Factor 3) explained 19.4% of the variance and the significant variables that were positively loaded in this factor represented different aspects of horizontal movement in the two areas of the OF (center and border). This suggests that this factor likely reflected general area locomotion. Factor 4 represented 16.1% of the variance and the significant variables positively loaded in this factor corresponded to behaviours in the center zone of the OF. This indicates that this factor characterized behaviour in the exposed zone of the open field test (center). Finally, Factor 5 accounted for 7% of the variance and was positively loaded by variables corresponding to behaviours in the open arms of the EPM, with a corresponding negative loading in the closed arm-related component. This factor was considered to be mainly characterizing behaviour in the exposed area of the EPM.

To extend our understanding of the behavioural data structure, we analysed the distribution of the behavioural variables in bi-dimensional spatial representations of the first three factors (dimensions) (Fig. 4B,C). These analyses demonstrated that behavioural variables were aggregated spatially along the factor axes (ellipses), reflecting their close relatedness. Furthermore, this representation revealed that indeed Factor 1 discriminated between absence (high negative loadings, green ellipse) and presence (high positive loadings, green ellipse) of horizontal movement (features associated with locomotion structure). On the other hand, Factor 2 differentiated relevant behaviours between open arms (high positive loadings, gray ellipse) *versus* closed arms (high negative loadings, grey ellipse) in the EPM test (reflecting risk/unconditioned fear). Finally, Factor 3 allowed the distinction between movement description characteristics (such as distance travelled or speed, blue ellipse) and actual behaviours in the center of the OF (high negative loadings, blue ellipse).

After the above description of the structure underlying the behavioural data, we next examined the spatial distribution of individual animals in the tridimensional space defined by Factors 1, 2 and 3. This was done by plotting each animal’s scores in each of the first three dimensions/factors (Fig. 4D). We observed distinct spatial distributions within each group and we next determined whether it would be possible to discriminate the three experimental groups based on the individual scores on the five identified factors (Fig. 4E). Our analysis showed similar scores between experimental groups for Factors 1, 3, 4 and 5. Strikingly, both TgWT and TgAaaH2KO-infected animals displayed significantly different loadings, relative to controls, for Factor 2, which represented the structure of risk/unconditioned fear behaviour. However, no differences were detected between the two parasite infected groups.

The results described above demonstrate mathematically that the scored behaviours are structured along specific dimensions (factors) and that parasite infection mainly results in deviations from normality in the risk/unconditioned fear behaviour, in an AaaH2 TH-independent way.

### Chronically infected animals are sensitive to trapping but react inadequately, irrespective of infective parasite strain

The behavioural data collected in the EPM allowed us to demonstrate that parasite infection (with either TgWT or AaaH2 TH mutant strains) resulted in changes in risk/unconditioned fear behaviour (mathematically corroborated by the factor analysis described above). This is in accordance with previous reports that *Toxoplasma* infection renders animals more susceptible to being trapped^7^. We therefore designed a test where we could submit animals to trapping events in the laboratory, and observe the subsequent changes in behaviour.

**Figure 5:**
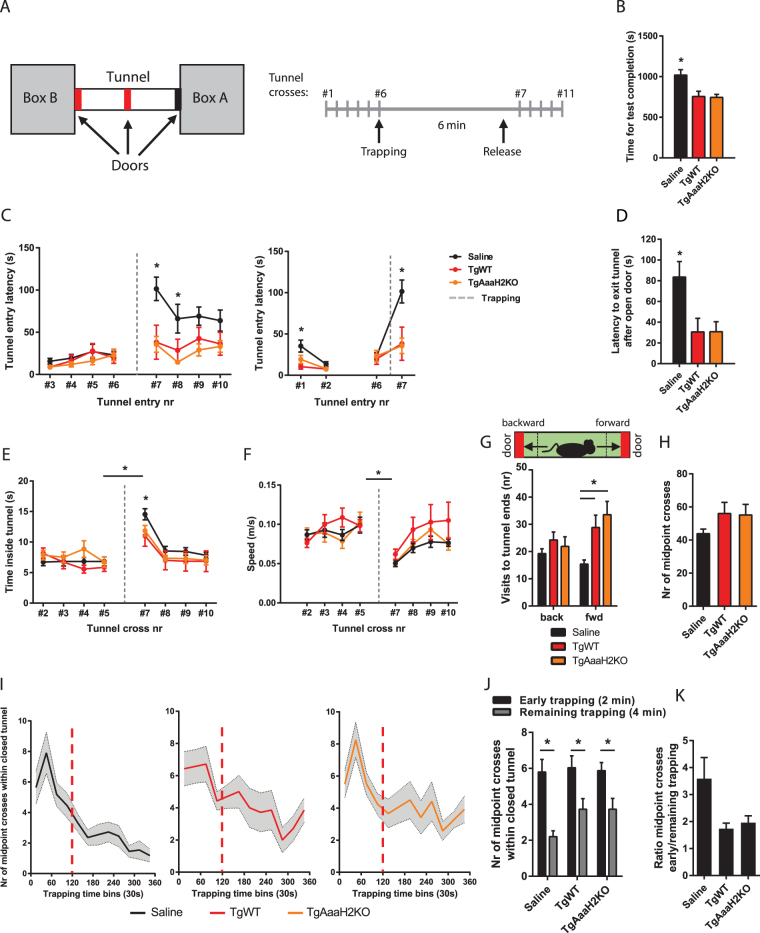
*Toxoplasma* infection of mouse hosts results in abnormal reaction to trapping, independently of parasite TH. (**A**) Schematic representation of the trapping behavioural setup. Two boxes were connected by one transparent tunnel, with three access doors indicated in red and black (left panel). Trapping occurs when the doors indicated in red were closed. The right panel describes the protocol for the tunnel trapping test (see Methods). (**B**) Infected animals were faster in completing the tunnel test when compared to controls. (**C**) Control animals showed higher latencies to re-enter the tunnel after trapping had occurred whereas no such behaviour was observed in infected animals (left panel). The latencies for first (#1) tunnel entry were lower in infected animals relative to controls, with this difference being lost on the second (#2) tunnel entry. Similar latencies for entry #6 (which precedes trapping) were observed in all experimental groups. However, after trapping, infected animals did not show the increase in latencies for tunnel entry #7 (first entry after trapping), observed in control animals (right panel). (**D**) After the trapping time ended and doors opened, infected animals exited the tunnel faster than control animals. (**E**) All experimental groups displayed an increase in the time spent per cross after being trapped but infected animals were faster than controls in the first cross after the trapping event (entry #7). (**F**) The reduction in speed observed as a response to the trapping event remained unchanged by infection. (**G**) During the trapping period, infected animals moved forward more often than controls. Virtual spatial thresholds for scoring visits to the ends of the enclosed tunnel section are indicated by the dashed vertical lines. (**H**) Infection did not alter the number of times animals crossed the enclosed section midpoint, during the trapping period. (**I**) Over time analysis of the distribution of midpoint crosses during trapping showed a more uniform movement profile in animals infected with the wild type parasite strain. Profiles were divided in 2 min and 4 min periods (red line) defining early and remaining trapping periods. (**J**) The decrease in animal movement from early to remaining periods in trapping was present in all infected animal groups. (**K**) When trapped, infected animals showed a more uniform movement over time, as suggested by a ratio decrease. (**B**,**C**,**E**,**F**,**H**–**K**) Mouse saline group, n = 11; TgWT group, n = 7; TgAaaH2KO group, n = 13. (**D**) Mouse saline group, n = 7; TgWT group, n = 6; TgAaaH2KO group, n = 9. (**G**) Mouse saline group, n = 8; TgWT group, n = 7; TgAaaH2KO group, n = 9. Dashed grey lines indicates trapping event. *p < 0.05. The data are presented as the mean ± SEM.

Animals were placed in one of two boxes connected by a tunnel (Box A, Fig. 5A, left panel) and tunnel access was granted by opening all dividing doors. Animals crossed the tunnel 5 times until two of the doors were closed (middle door and tunnel end door), trapping the animal inside a small section of the tunnel. After a 6-minute trapping period, animals were released by opening the doors (Fig. 5A, right panel, see Materials and Methods). This experimental design permitted within-animal comparison of the behaviour before and after the trapping event.

Infected animals were faster in completing the entire test (5 crosses followed by a 6 min trapping period and execution of 5 additional crosses) (Fig. 5B). Additionally, analysis of the time that control animals took to re-enter the tunnel after trapping showed a latency increase in the first entry immediately after trapping (entries #6 *vs* #7, Fig. 5C, left panel). This is consistent with a reluctance to re-enter the space where trapping was experienced. The increase in latency to enter the tunnel after trapping was not observed in infected experimental groups (independently of parasite strain), with animals displaying similar (low) latencies throughout the test. This lower entry latency relative to controls was observed in infected animals as early as the very first tunnel entry (Fig. 5C, right panel).

Once the trapping period was over, the doors opened, releasing the animal. Interestingly, infected animals exited the tunnel faster than control animals (Fig. 5D), only to re-enter it faster too (Fig. 5C, left panel). We also compared how much time animals spent in each cross, before and after trapping (Fig. 5E). All experimental groups showed an increase in this parameter after trapping, indicating that all groups, irrespective of infection status, were sensitive to the trapping event, and reacted to it by taking longer to cross the tunnel. However, infected animals were faster than controls in the first cross after trapping (entry #7). Interestingly, the tunnel crossing speed was similar between all groups, with an equivalent reduction in speed following trapping (Fig. 5F).

During the 6-minute trapping period, animals were only able to move forward or backward within the confined space of the tunnel. We quantified the number of visits to each end of the tunnel (reflecting forward and backward movements) and infected animals displayed a higher drive to move forward than controls (Fig. 5G). To extend the analysis of animal movement during trapping, we defined a point halfway through the enclosed tunnel section and scored how many times animals crossed this point (midpoint). This allowed for the tracking of movement over trapping time. Overall, no statistically significant difference between groups was found in total number of enclosed section midpoint crosses (Fig. 5H). However, this metric does not capture finer movement dynamics so we next analysed how the number of midpoint crosses were distributed over time. All animals displayed high movement, which then decreased as time progressed (Fig. 5I). To further characterize this reduction, we divided the trapping time into two periods: early (first 2 minutes of trapping period) and the remaining trapping time (4 minutes) and scored the number of midpoint crosses in these two periods. Overall, we found that animals in all experimental groups did indeed decrease their movement as trapping time elapsed (Fig. 5I and J), but the movement plots also suggested that this reduction evolved differently over time in infected animals. To clarify this, we determined the magnitude of the reduction in movement by calculating the ratio of number of midpoint crosses in early trapping *versus* the remaining trapping time (Fig. 5K). Although statistical analysis of this calculation did not reach significance (Table S1), it showed that infected animals have a smaller ratio relative to controls (reduction to half of the control values). This smaller ratio reflects a more uniform, constant movement throughout trapping time.

Overall, these data demonstrate that *Toxoplasma*-infected animals react differently to a trapping event compared to control animals. These findings suggest that infected animals might be more likely trapped in real world environments and the underlying behavioural changes do not require AaaH2 TH to be present in the parasite.

## Discussion

In this study, we tested the involvement of parasite-derived AaaH2 TH in the behaviour modifications that occur in *Toxoplasma* chronic mouse infection. We removed the *AaaH2* gene from the parasites’ genome and demonstrated that this deletion has no impact in expression levels of the paralog *AaaH1* gene. Additionally, we established that the progression of mouse infection over time is indistinguishable between parental and mutant parasite strains. Moreover, AaaH2 TH deletion did not prevent cyst formation, demonstrating that this enzyme is not necessary for *in vivo* cyst differentiation, consistent with recent reports for cyst growth in *in vitro* and *in vivo* conditions^21,22^. In fact, one recent study demonstrates that AaaH2 TH does have a role in the parasite’s life cycle but in oocyst development within the cat host and may not relevant for infection of intermediate hosts (such as mice)^22^.

We compared the behaviour of animals chronically infected with both parasite strains using two distinct behavioural paradigms. Analysis of exploratory locomotion revealed that mice infected with the mutant parasite strain still exhibited behaviour changes. These changes were comparable to those induced by parental strain infection, with animals moving more, faster and reducing the number of shorter bouts in favour of longer ones. Additionally, all infected animals behaved similarly in relation to novel, exposed areas, with a general loss of the cautious behaviour observed in control animals, as indicated by the reduction of unconditioned fear and increase in riskier behaviours. All of these very robust behaviour modifications were generated despite the overall low numbers of brain cysts. This demonstrates that behavioural modifications following parasite infection do not depend on high cyst numbers and further supports the idea that this is not a unidimensional phenomenon.

We extended the behaviour analysis to establish that behaviours were clustered along five specific dimensions/factors. Furthermore, we demonstrated that infected mice were different from non-infected animals in a single factor, which represented the structure of risk/unconditioned fear behaviour, independently of infective strain. It would be expected that the changes in exploratory locomotion, observed in infected animals during the open field test (see Fig. 2), would also be reflected in this mathematical analysis. One would expect that scores for the factor which represents the structure of exploratory locomotion would allow discrimination of infected from non-infected experimental groups. However, no differences were found, suggesting that the modifications in locomotion mostly reflect a general reduction in the risk/unconditioned fear behaviours (and not other locomotor alterations like hyperactivity, for example).

All the data presented so far demonstrate that mice chronically infected with *Toxoplasma* parasites will behave differently in exposed environments, deviating from the expected cautious/defensive behaviour. However, it has not been possible to determine whether these changes stem from a distorted perception of risk in the environment or from an inability of infected animals to adjust the behavioural responses appropriately. To clarify this, we developed a new behavioural paradigm in which animals were subjected to an unpredictable external event (trapping), within the same single test, and their resulting behaviour was observed. We showed that all animals can perceive the event as aversive since individuals in all three experimental groups took longer to cross the tunnel after being trapped. However, infected animals were not able to modulate further entries, showing no differences in entry latencies before and after trapping, whereas control animals were reluctant to re-enter the tunnel after trapping. Additionally, infected animals completed this test faster by, following trapping, exiting the tunnel quicker and re-entering it sooner. When trapped, these animals also moved forward more frequently and showed more uniform movement over time. This indicates that infected animals are actually in a different behavioural state that may reflect a drive to maintain forward movement. This is, to our knowledge, the first demonstration that infected animals are sensitive to an unpredictable challenge in the environment, but show difficulty in adjusting their behaviour afterwards.

The present study demonstrates that parasite-secreted TH (resulting from *AaaH2* gene expression) is not necessary for the generation of the host behaviour modifications observed in *Toxoplasma* chronic infection. This fact makes it hard to argue that the ability for the parasite to manipulate dopamine metabolism is the one mechanism through which these changes occur. There is mounting evidence that chronic infection impacts multiple physiological dimensions in the host organism, not only locally (neuronal structure) but also more globally (peripherally-triggered immune responses)^23^. These local and global effects can directly or indirectly impact the regulation of neurotransmitters other than dopamine (serotonin, GABBA, glutamate) and, consequently, neuronal function. It seems likely then that the host behavioural phenotypes observed in *Toxoplasma* chronic infections are the net products of a complex interplay between distinct processes which might not involve dopamine dysregulation.

All of the considerations above highlight the complexity of behaviour manipulation following parasitic infection and the need for studies which integrate different biological dimensions. Clarification of their mechanistic contributions to host behaviour is therefore fundamental. It will also be important to determine whether these behaviour modifications are present in mouse models more resistant to parasitic infection and even in other known rodent hosts, such as rats.

## Methods

### Ethics statement

All experimental procedures were reviewed and approved by the Animal Welfare Body (ORBEA) and reviewed by the Portuguese Veterinary General Board (Direcção Geral de Veterinária, approval ID Ref. No. 0421/000/000/2014), in accordance with FELASA (Federation of European Laboratory Animal Science Associations)’s recommendations regarding the care and use of laboratory animals.

### Mouse and parasite strains

These studies used female C57BL/6 J mice (The Jackson Laboratory, Maine, USA), aged 7 weeks.

A genetically modified type II strain of *Toxoplasma gondii* (ME49^*BAG1/EGFP*^) was used, expressing green fluorescent protein (GFP) driven by the cyst-stage specific BAG1 promoter, as previously described^24^. To generate the vector for the *AaaH2* gene deletion in the ME49^*BAG1/EGFP*^ strain background, we first amplified fragments of 3070 and 2995 bp upstream and downstream of the ATG and STOP codon of the *AaaH2* gene, respectively, using the *Phusion* polymerase (New England Biolabs). The primers used to amplify the 5′ sequence/arm were F1 KpNI (5′GACTGGTACCAAGATAGAGTGGCGTATGTGTCCAAGC3′) and R1 HindIII (5′GACTAAGCTTGAATGCGTTACGGTGATCGGAGACATAC3′). To amplify the 3′ sequence/arm, the following primers were used: F2 BamHI (5′GACTGGATCCAGACACTGTATCTCTACTTTGCATTC3′) and R2 NotI (5′GACTGCGGCCGCGAATTCGGATGAGTCACCCAGTGCATGC3′). The 5′- and 3′-arms were then cloned upstream and downstream of the dihydrofolate reductase protein (DHFR) expression cassette, respectively, using the *KpN*I/*Hind*III and *BamH*I/*Not*I restriction sites^25^. The selectable marker DHFR confers resistance to the drug pyrimethamine. In order to identify and exclude parasites with the *AaaH2* knockout cassette integrated by single instead of the double crossover (which is necessary for full *AaaH2* gene deletion), we cloned a tubulin/EGFP cassette upstream of the *AaaH2* 5′arm using the *ScaI*/*KpNI* restriction sites. If a single 5′ integration event occurs (and not the intended 5′-3′ double integration), green fluorescent tubulin will be expressed. Before transfection, the linear DNA fragment tubulin*/EGFP_5*′*ARM*_*Aaah2*_*-DHFR-3*′*ARM*_*Aaah2*_ was isolated following *SacII* restriction enzyme digestion.

Fifty µg of the *SacII* digested pAaaH2 plasmid were used to transfect 2 × 10^7^ ME49^*BAG1/EGFP*^ tachyzoites as described in ref.^26^. After pyrimethamine selection and cloning, 300 single parasite clones were analysed by fluorescence microscopy and 34 tubulin/EGFP negative clones were selected for further analysis. Purified genomes of the selected parasites were analysed by PCR and one was identified (TgAaaH2KO) as having the *AaaH2* gene deleted and the expected integration of the DHFR cassette in the target genomic locus. Primer sequences used are listed in Supplementary Table S2 (Supplementary Information).

### Host cells and parasite culture

Human foreskin fibroblasts (HFF; Hs27, CRL-1634^TM^, ATCC) or Vero cells (green monkey kidney cells, CCL-81^TM^, ATCC) were grown as described elsewhere^9^ and both parasite strains were maintained as tachyzoites and propagated *in vitro* by serial infection of cell monolayers.

### Quantitative real-time PCR procedure

The TgAaaH2KO and parental strains were allowed to infect HFF monolayers and i*n vitro* differentiation of TgAaaH2KO and parental strain bradyzoites was achieved by alkaline conversion for 7 days. This was then followed by pepsin treatment as described in ref.^27^ and total RNA extraction with Trizol reagent. Bradyzoite conversion was confirmed by checking for GFP expression driven by the cyst-stage specific BAG1 promoter.

Total RNAs were treated with RNAse-free DNase I (New England Biolabs) for 30 min at 37 °C to remove genomic DNA contaminants. After phenol/chloroform extraction and ethanol precipitation, 5 µg of RNA were reverse transcribed using the SuperScript IV VILO Master Mix (Thermo Fisher), according to the manufacturer’s instructions. Two microliters of cDNA from each sample were used to run quantitative real-time PCR (qPCR) using the SYBR® Select Master Mix (Thermo Fisher), using primers for *Toxoplasma* tubulin sequence, as reference housekeeping gene (TUBrtFW1 5′GCGTCTTCT TGGATTTGGAG3′ and TUBrtRV1 5′TGGAGACCAGTGCAGTTGTC3′) or for the *AaaH1* gene (AaH1rtFW1 5′CCACAAGCCTTT CGCAACGAGATATGAC3′ and AaH1rtFW2 5′CTCCACACACACGCA TCTTCGCACATTG3′) and the StepOne Real-Time PCR Systems equipment (Thermo Fisher). The thermal cycling conditions consisted of an initial step at 50 °C for 2 min, followed by a denaturation cycle at 95 °C for 2 min, 40 cycles at 95 °C for 15 sec and 60 °C for 1 min. After qPCR amplification, the equipment was programmed to perform a melting curve. The specificity of results of real-time RT-PCR was confirmed by agarose gel electrophoresis of amplicon products, checking for the existence of single band of expected size, and by qPCR analysis of negative controls (no template and no reverse transcriptase). Relative gene expression analysis was performed using the comparative Ct method (2^−∆Ct^) and fold changes were calculated using the comparative ∆Ct method (2^−∆∆Ct^)^28^. All samples were run in biological and technical triplicates.

### Plaque assay

Intracellular tachyzoites of TgAaaH2KO or parental strain were mechanically extruded from human foreskin fibroblasts, by repeated passage of scraped infected monolayers through a 26 G needle. One hundred freshly extruded tachyzoites were added to each well of six-well plates containing HFF monolayers and incubated undisturbed at 37 °C and 5% CO_2_. After 12 days, infected monolayers were fixed in methanol for 2 minutes, stained with Crystal violet (Sigma HT90132) and plaques were counted.

### Animal infection

Female mice were first weighed and then inoculated intraperitoneally using 1 ml syringes (29 G, 0.33 mm × 12.7 mm). The parasite load used was 10 000 tachyzoites/animal, resulting in two experimental infection groups: TgWT- and TgAaaH2KO-infected animals. The control group consisted of saline (PBS) injected mice. After injection, animals were housed four per cage and evaluated weekly for general health and weight status. The behavioural tests were performed eight weeks post-infection and experimental results were obtained from pooled data from three separate experiments.

In the present report, all parasite-injected animals developed a sickness phenotype, distinctly from what was reported in our previous study^9^, where some animals did not show any clinical phenotype or develop brain cysts, despite being injected with parasites. Since the experiments in the present study were conducted in a different research facility, this could be due to differences in animal immune status, mouse colony or general vivarium conditions.

### Histological preparation

Once all behavioural tests were completed, mice were sacrificed by first inducing anaesthesia with isofluorane, followed by intraperitoneal injection with Ketamine/Xylaxine (~5 mg/Kg xylazine; 100 mg/kg ketamine). Mice were perfused with PBS and 4% paraformaldehyde, and brains were extracted. Cryoprotection of brain tissue was achieved by overnight immersion 30% sucrose:PBS solution (Sigma) and each brain was coronally sectioned in 40 µm slices, by using Leica cryostat CM3050S, set at −25 °C.

### Immunohistochemistry

Immunohistochemistry of infected brain slices was performed to increase the fluorescence signal in parasite cysts. After washing with PBS, sections were incubated for 10 min at room temperature in 0.1 M Glycine:PBS. This was followed by a 10 min (room temperature) permeabilization step with 0.5% Triton X-100:PBS. Next, a blocking step (30 min, room temperature, with 10% Foetal Bovine Serum:0.2% Triton X-100:PBS) preceded the incubation of tissue sections with the anti-GFP antibody conjugated with Alexa 488 fluorochrome (1:500, rabbit polyclonal, Molecular Probes), 1 h at room temperature. Sections were washed and DNA was counterstained with 4′,6-diamidino-2-phenylindole (DAPI, Sigma). Finally, sections were mounted in MOWIOL (4–88 Reagent, Merck) and sealed with nail polish. Visualization of sections was performed using a wide field fluorescence microscope (AxioImager, Zeiss) and cysts were scored in all brain slices. Cyst quantification was performed in the brains of animals infected in two (TgWT) or three (TgAaaH2KO) separate experiments.

### Behavioural setups

Animal housing was performed under a 12 h light-dark cycle (lights on at 8 a.m.), food and water being available *ad libitum*. The behavioural tests were executed between 8 a.m. and 1 p.m. and the setups were cleaned between each animal test with 10% ethanol:PBS.

Mice were tested with 24 h intervals and test order was: open field, elevated plus maze and tunnel setups. Daily testing was performed blind and mixed groups were formed, composed of individuals from each of the 3 experimental conditions (saline, TgWT- and TgAaaH2KO-infected animals). All animals were exposed to each behavioural setup only once.

The open field consisted of a square acrylic arena with white floor and black walls (39.5 × 39.5 × 17.5 cm). This setup was enclosed in a black cloth covered area, so that any visual cues were uniform. Each mouse was placed in the middle of the arena and allowed to freely explore for 10 min. Fifty percent of the available area was defined as the center zone. Animal movement was captured by using a Sony Handycam DCR-SR57E at 25 frames/second.

The elevated plus maze acrylic arms were black (walls) and white (floor), 39.5 cm long and 5 cm wide. Two opposing arms were surrounded by 15 cm high walls and each arm was attached to a supporting structure, 40 cm away from the floor. Illumination was uniform in both open and closed arms. Each animal was placed at the intersection of the four arms (always facing an open arm) and allowed to explore for 5 min. Movement was recorded using a Sony Handycam DCR-SR57E (25 frames/second).

The current tunnel setup was first described in^29^ and was composed of two acrylic boxes (12 cm × 8 cm × 12 cm), connected by a transparent acrylic tunnel (48 cm × 3 cm × 3 cm; internal dim 2.5 cm × 2.5 cm). Access to the tunnel was controlled by mechanically operated doors (one at each end of the tunnel and an additional one in the middle). The test started with each animal being placed in the same starting box (Box A) and the tunnel access door would then open. Individuals were allowed to cross the tunnel 5 times and, on the 6^th^ cross, the doors (middle door + door of the box the animal had just exited from) would close and trap the animal inside the tunnel, for 6 min. After this time elapsed, the doors would open and release the animal. The test would end when another 5 consecutive crosses were executed. Behaviour was recorded using a PointGrey Flea3 camera at 30 frames/second.

### Behavioural data analysis

Video behavioural data recorded from the open field and elevated plus maze setups was automatically analysed using Anymaze software (Stoelting, USA). Behavioural data obtained in the tunnel setup was either scored by an experimenter blinded to the animals’ identities or analysed using a custom written code in Matlab programming language (MathWorks). Graphics were plotted using Graphpad Prism (GraphPad Software, Inc., USA).

In the open field data set, the coefficient of variation plot in Fig. 2D was calculated based on the distance travelled in 2 min intervals, for each individual, dividing the standard deviation by the average distance travelled over time. The bouts of movement in Fig. 2E plot were defined as the number of horizontal transitions from immobility to mobility and back. The frequency of bout occurrence (Fig. 2G) was scored as the proportion of bouts of particular length intervals. Non-locomoting periods (Fig. 2K) correspond to episodes where no horizontal movement is detected.

In the elevated plus maze data set, occupancy (Fig. 3D) was calculated as the percentage of time spent in each arm type. Occupancy of the center of the maze was similar across all experimental groups and was not represented.

Additional behaviour variables were scored in the open field and elevated plus maze tests and used for principal component analysis (see below) and are listed in column 2 in Fig. 4. Open field: Time immobile (center and border) corresponded to the average percentage of time immobile in a given area relative to total time spent in that area. Time freezing was calculated as the average percentage of time the animal spent freezing (absence of motion apart for respiratory-related movements) relative to test duration. Time freezing in center was the proportion of time spent freezing in center relative to time spent on center. The percentage of time spent in center relative to test duration was designated as center occupancy.

In the tunnel trapping test, visits to tunnel ends (either by the animal moving forward or backward) were scored as crosses of a virtual line positioned 5 cm away from each tunnel end. The number of tunnel midpoint crosses was calculated by defining a point halfway through the enclosed tunnel section and scoring the number of times animals crossed this point (midpoint). The distribution of midpoint crosses over trapping time was analysed in early (first 2 minutes of trapping period) and the remaining trapping time (4 minutes) and the number of crosses within each period was calculated for 30 s bins.

### Statistical analysis

Statistical analyses were carried out using R studio softwate (Rstudio, Inc.) and Graphpad Prism (GraphPad Software, Inc., USA) and all data are represented as mean ± SEM.

Data distribution was first tested for normality (using the Shapiro test) and for variance homogeneity (Levene’s test). Data not violating normality was analysed using independent samples tests, one-sample test (compare mean to value of 1), one-way ANOVA or two-way mixed design ANOVA, followed by the Bonferroni *post hoc* test. For data not conforming to homogeneity of variance, Welch correction was used for the ANOVA analysis, followed by robust *post hoc* test mcppb20^30^. Both the confidence intervals and p-values are corrected for the number of tests. Data that did not conform to normality was analysed using the Kruskal-Wallis rank-sum test and *post hoc* tests correspond to performing Wilcoxon rank-sum tests on all possible comparisons. This method is described in ref.^31^ and compares the difference between the mean ranks of groups to two values: one based on the value of z (corrected for the number of comparisons being done) and a constant calculated using the total sample size and the sample size in the two groups under comparison. Supplementary Table S1 (Supplementary Information) shows the results for each relevant statistical test.

Multivariate factor analysis was used to decrease data complexity and reveal the structure within the data sets with as little information loss as possible. We first standardized all behaviour data by calculating individual’s z-scores relative to the overall average value of the three experimental groups and principal component analysis (PCA) method was applied (involving a Varimax (orthogonal) rotation) to 26 of 30 behaviour variables scored in the open field and elevated plus maze experiments (sample sizes: saline = 28; TgWT = 15; TgAaaH2KO = 25). The data rotation used allowed for the maximization of variance within each extracted factor and thus resulted in a simpler structure interpretation. The analysis adequacy was determined using the Kaiser-Meyer-Olkin measure of sampling adequacy test (KMO = 0.82) and the Bartlett’s sphericity test. The 5 identified factors with loadings >0.40 represent the primary dimensions being measured and account for 84.3% of all variance present in de data. An Anderson-Rubin factor score matrix was calculated, representing the classification of each animal on the identified factors.

### Data Availability

The datasets generated during and/or analysed during the current study are available from the corresponding authors on reasonable request.

## Electronic supplementary material

Supplementary Information
