# Supplementary material for: Toxoplasma-induced changes in host risk behaviour are independent of parasite-derived AaaH2 tyrosine hydroxylase
Source: Sci Rep. 2017 Oct 23;7:13822. doi: 10.1038/s41598-017-13229-y (PMC5653819; doi:10.1038/s41598-017-13229-y)

Toxoplasma-induced changes in host risk behaviour are independent of parasite-derived AaaH2 tyrosine hydroxylase

Cristina Afonso[1], Vitor B. Paixão[1], Andreas Klaus[1], Matteo Lunghi[2], Federica Piro[2], Carla Emiliani[2], Manlio di Cristina[2]*, Rui M. Costa[1]*

[1] Champalimaud Center for the Unknown, Champalimaud Neuroscience Programme, Av. Brasília, Doca de Pedrouços - 1400-038 Lisboa, Portugal

[2] University of Perugia, Department of Chemistry, Biology and Biotechnology, Building B, Via del Giochetto, 06122 – Perugia, Italy

* Corresponding author (Manlio di Cristina - manlio.dicristina@unipg.it ; Rui M. Costa - rui.costa@neuro.fchampalimaud.org)

Supplementary Table S1 – Relevant statistical results.

Figures	Statistical test	Results
**1E**	Mixed model	Main effect week pI (within subjects) χ^2 (8)=235.0, *p*<0.05; Main effect treatment group (between subjects) χ^2 (2)=83.18, *p*<0.05; Interaction effect χ^2 (16)=384.6, *p*<0.05.
**1F**	One-way ANOVA	F(2,65)=38.58, *p*<0.05; post hoc tests significant in saline *vs* TgWT groups and saline *vs* TgAaaH2KO groups.
**1G**	Independent samples t-test	t-test t(17.94)=3.44, p<0.05.
**2A**	Kruskal-Wallis rank sum test	H(2)=12.27, *p*<0.05; post hoc tests significant in saline *vs* TgWT groups and saline *vs* TgAaaH2KO groups.
**2B**	Kruskal-Wallis rank sum test	H(2)=10.34, *p*<0.05; post hoc tests significant in saline *vs* TgWT groups and saline *vs* TgAaaH2KO groups.
**2C**	Two-way repeated measures ANOVA	Main effect treatment group F(2, 65) = 4.93, *p*<0.05; main effect time F(4, 260) = 38.10, *p*<0.05; interaction effect F(8,260)=3.92, *p*<0.05; post hoc tests significant between treatment groups for mins 6 and 10.
**2D**	One-way ANOVA	F(2,65)=7.82; post hoc tests significant in saline *vs* TgWT groups and saline *vs* TgAaaH2KO groups.
**2E**	One-way ANOVA	F(2, 65) = 3.58; post hoc tests significant in saline *vs* TgWT groups.
**2G**	Two-way repeated measures ANOVA	Main effect bout length F(7,455)=39.17, *p*<0.05; interaction effect F(14,455)=2.27, *p*<0.05; post hoc tests significant between treatment groups for bout lengths [0-5]s, [11-20]s and [41-80]s.
**2H**	Two-way repeated measures ANOVA	Main effect treatment group F(2,65)=4.80, *p*<0.05; main effect OF area F(1,65)=70.69, *p*<0.05; interaction effect F(2,65)=3.91, *p*<0.05; post hoc tests significant in saline *vs* TgAaaH2KO groups in border.
**2I**	Two-way repeated measures ANOVA<br><br>Center only – Kruskal-Wallis rank sum test (H(2)=9.08, *p*<0.05; post hoc tests significant in saline *vs* TgWT groups and saline *vs* TgAaaH2KO groups)	Main effect treatment group F(2,65)=4.43, *p*<0.05; main effect OF area F(1,65)=171.8, *p*<0.05; post hoc tests significant in saline *vs* TgAaaH2KO groups in center.
**2J**	Two-way repeated measures ANOVA	Main effect treatment group F(2,65)=4.23, *p*<0.05; interaction effect F(2,65)=3.39, *p*<0.05; post hoc tests significant in saline *vs* TgWT and saline *vs* TgAaaH2KO groups in center.
**2K**	Two-way repeated measures ANOVA	Main effect OF area F(1,65)=62.8, *p*<0.05; post hoc tests significant in saline *vs* TgAaaH2KO groups in center.
**3A**	Robust Wilcox ANOVA	F(2,18.82)=4.06; post hoc tests significant in saline *vs* TgWT groups.
**3B**	Robust Wilcox ANOVA	F(2,22.93)=7.02; post hoc tests significant in saline *vs* TgWT groups and saline *vs* TgAaaH2KO groups.
**3C**	Two-way repeated measures ANOVA	Main effect EPM arm F(1,65)=56.17, *p*<0.05; main effect treatment F(2,65)=3.29, *p*<0.05; post hoc tests significant in saline *vs* TgWT and saline *vs* TgAaaH2KO groups in open arms.
**3D**	Two-way repeated measures ANOVA	Main effect treatment group F(2,65)=5.51, *p*<0.05; main effect EPM arm F(1,65)=7.68, *p*<0.05; interaction effect F(2,65)=6.39, *p*<0.05; post hoc tests significant in saline closed arm vs open arms.

**3E**	Two-way repeated measures ANOVA	Main effect EPM arm $F(1,65)=11.67$, $p<0.05$; post hoc tests significant in saline closed arm vs open arms.
**3F**	Kruskal-Wallis rank sum test	$H(2)=9.17$ $p<0.05$; post hoc tests significant in saline *vs* TgWT groups.
**4E**	One-way ANOVA within each PCA factor (F1-F5)	$F(2,65) = 3.35$; post hoc tests significant in saline *vs* TgWT groups for factor F2.
**5B**	One-way ANOVA	$F (2, 27) = 8.69$; post hoc tests significant in saline *vs* TgWT groups and saline *vs* TgAaaH2KO groups.
**5C (left panel)**	Two-way repeated measures ANOVA	Main effect number of entry $F(7,203)=11.41$, $p<0.05$; main effect treatment $F(2,29)=11.09$, $p<0.05$; interaction effect $F(14,203)=2.84$, $p<0.05$; post hoc tests significant in saline *vs* TgWT and saline *vs* TgAaaH2KO groups for entries #7 and #8.
**5C (right panel, entries #1-#2)**	Two-way repeated measures ANOVA	Main effect number of entry $F(1,29)=9.38$, $p<0.05$; main effect treatment $F(2,29)=5.94$, $p<0.05$; post hoc tests significant in saline *vs* TgWT and saline *vs* TgAaaH2KO groups for entry #1.
**5C (right panel, entries #6-#7)**	Two-way repeated measures ANOVA	Main effect number of entry $F(1,29)=22.35$, $p<0.05$; main effect treatment $F(2,29)=5.42$, $p<0.05$; interaction effect $F(2,29)=8.49$, $p<0.05$; post hoc tests significant in saline *vs* TgWT and saline *vs* TgAaaH2KO groups for entry #7.
**5D**	One-way ANOVA	$F (2, 19) = 5.93$; post hoc tests significant in saline *vs* TgWT groups and saline *vs* TgAaaH2KO groups.
**5E**	Two-way repeated measures ANOVA	Main effect number of entry $F(7,203)=26.66$, $p<0.05$; interaction effect $F(14,203)=2.43$, $p<0.05$; post hoc tests significant for entry #5 *vs* entry #7 in all experimental groups; post hoc tests significant in saline *vs* TgWT groups for entry #7.
**5F**	Two-way repeated measures ANOVA	Main effect number of entry $F (8, 224) = 11.78$, $p<0.05$; post hoc tests significant for entry #5 *vs* entry #7 in all experimental groups.
**5G**	Two-way repeated measures ANOVA	Main effect tunnel extremity $F (1, 21) = 5.40$, $p<0.05$; interaction effect $F (2, 21) = 6.19$, $p<0.05$; post hoc tests significant for forward visits in saline *vs* TgWT groups and saline *vs* TgAaaH2KO groups.
**5J**	Two-way repeated measures ANOVA	Main effect time of trapping period $F (1, 27) = 42.77$, $p<0.05$; post hoc tests significant in saline, TgWT and TgAaaH2KO groups for early *vs* remaining trapping period.
**5K**	One-way ANOVA	$F (2,27) = 3.57$; post hoc test significant only without correction for multiple comparisons (using Fisher's LSD test) for saline vs TgWT and saline vs TgAaaH2KO ($p=0.03$).

Supplementary Table S2 – Primers sequences used for PCR analysis for identification of TgAaaH2KO clone used in this study.

P1 - CGCAGCAGACAAAAGTGCAAGGCAGAAC
P2 - GGGAGACAGGAGGCATATGT
P3 - CATTCGGCACCACTCGGCTCCTCTG
P5 - CAACTGATGTACATATGCCT
P6 - GTCTCAATATGGATACATACATG
P7 - ACAGTGTGTGCACTTGCACACCAGATTG
P8 - TGATTCCTGATCACTCCGCTGATCAGAG
P9 - CTATCAGTTGTTTTAGTCGAACCGGTTAAC
P10 - CACTACGCGAGAGAATATGCGAAGAAGC

Supplementary Figure S3 - Full-length gels corresponding to cropped portions in Figure 1B. Relevant lanes are boxed in white and primer pairs indicated above. M - molecular weight marker 1kb DNA ladder (NEB).

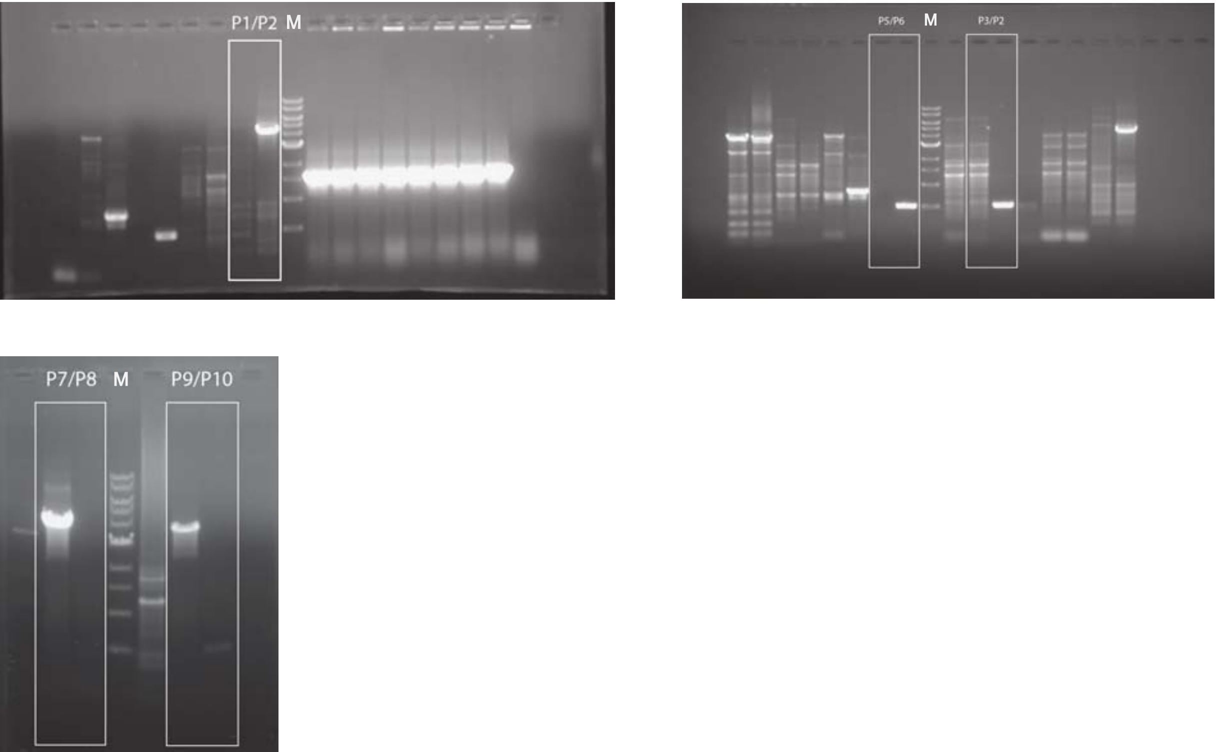

Supplement: Supplementary file 1 — Supplementary Information [file 41598_2017_13229_MOESM1_ESM.pdf]
